# 4-Nitrobenzene Grafted in Porous Silicon: Application to Optical Lithography

**DOI:** 10.1186/s11671-016-1654-8

**Published:** 2016-09-29

**Authors:** Mariavitalia Tiddia, Guido Mula, Elisa Sechi, Annalisa Vacca, Eleonora Cara, Natascia De Leo, Matteo Fretto, Luca Boarino

**Affiliations:** 1 Dipartimento di Fisica, Università di Cagliari, Cittadella Universitaria, S.P. 8 km 0.700, 09042 Monserrato, CA Italy; 2 Dipartimento di Ingegneria Meccanica, Chimica e dei Materiali, Università degli Studi di Cagliari, Via Marengo 3, 09123 Cagliari, CA Italy; 3 Nanofacility Piemonte INRiM (Istituto Nazionale di Ricerca Metrologica), Strada delle Cacce 91, 10135 Torino, Italy

**Keywords:** Porous silicon, Optical lithography, 4-Nitrobenzenediazonium grafting, Improved chemical resistance

## Abstract

In this work, we report a method to process porous silicon to improve its chemical resistance to alkaline solution attacks based on the functionalization of the pore surface by the electrochemical reduction of 4-nitrobenzendiazonium salt. This method provides porous silicon with strong resistance to the etching solutions used in optical lithography and allows the fabrication of tailored metallic contacts on its surface. The samples were studied by chemical, electrochemical, and morphological methods. We demonstrate that the grafted samples show a resistance to harsh alkaline solution more than three orders of magnitude larger than that of pristine porous silicon, being mostly unmodified after about 40 min. The samples maintained open pores after the grafting, making them suitable for further treatments like filling by polymers. Optical lithography was performed on the functionalized samples, and electrochemical characterization results are shown.

## Background

Porous silicon (PSi) has been studied for a wide variety of applications in many fields, spanning from gas- and bio-sensing to photonics and photovoltaics [1, 2].

Photolithography on Porous Si (PSi) after the formation of the porous layer is usually a difficult task for several reasons that mainly come from both the high reactivity of the porous layer and on unwanted pore filling [3]. PSi patterning is usually achieved by performing a lithographic process on silicon surface before the PSi electrochemical etching [4] or through holographic methods directly on the porous surface [5, 6]. It is well known that the optical lithography on PSi is hindered by the high reactivity of the material being easily etched by alkaline solutions [7]. On the other hand, literature reports several methods for the stabilization of the PSi surface, e.g., thermal carbonization, hydrosilylation, electrochemical modification, or high-temperature thermal oxidation [8–16]. Another method is the polymer impregnation of PSi that has been proposed a few years ago by L. De Stefano et al. [17] with interesting results in terms of layer stability. In that case, the impregnation was performed by drop casting and spin coating. Here, we propose a simple approach functionalizing the whole PSi surface by the electrochemical reduction of 4-nitrobenzendiazonium (4-NBD) salt. We demonstrate that the 4-NBD functionalization induces a remarkable resistance to alkaline solutions in PSi and making it appealing for further chemical and/or electrochemical impregnation with polymers.

**Fig. 1:**
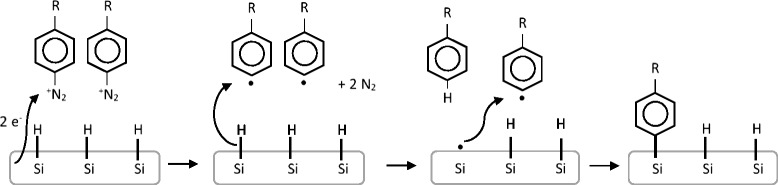
Mechanism of electrochemical grafting of aryl diazonium salts on H-terminated Si surface [20]

The grafting of organic monolayers on silicon surface is of great interest because it allows to obtain dense and ordered layers able to protect the surface or to confer it specific properties [18]. The first way proposed to promote Si-C covalent bond was by chemical or photochemical reactions: the hydrosilylation of alkenes at H-terminated Si surfaces and the silanization of hydroxyl-terminated Si surfaces are just some examples of the modification of silicon with organic compounds. An affordable route to obtain covalent grafting on Si surface is represented by the electrochemical reduction of diazonium salts: as proposed earlier in 1997 [19], 4-nitro- and 4-bromobenzenediazonium salts can be electrochemically reduced on H-terminated Si (111) surfaces in an aqueous acidic HF solution. The proposed reaction mechanism (see in Fig. 1) starts with the cleavage of dinitrogen and the formation of phenyl radicals which abstract a hydrogen atom from the surface to produce a silyl radical. The silyl radical reacts with a second phenyl radical, allowing the formation of a Si-C bond [20].

More recently, the reduction of different diazonium salts on Si has been demonstrated with no applied voltage due to the spontaneous electron transfer at the open circuit potential, which leads to the local generation of aryl radicals [21, 22]. The required contact time for the spontaneous grafting ranges from 30 to 60 min while the potential-driven electrochemical reduction allows faster reaction rates and the surface of silicon is protected against the formation of SiO_2_. It is worth to note that H-terminated silicon is not strictly necessary for the electrochemical reduction of diazonium salt: nitrobenzene and 4-methoxydiphenylamine have been grafted on Si surfaces with electrochemically grown SiO_2_ layers by K. Roodenko et al. [23].

## Methods

### Chemicals

Anhydrous acetonitrile (ACN, 99.8 %), 4-nitrobenzenediazonium tetrafluoroborate (4-NBD salt), and tetrabutylammonium hexafluorophosfate (TBAPF6) were purchased from Sigma-Aldrich®. Hydrofluoric acid (HF, 50 %) and sodium hydroxide (NaOH, pellets) were purchased from Carlo Erba Reagents Srl.

### Fabrication of PSi and Functionalization with 4-NBD

PSi was prepared by using highly phosphorous-doped Si wafers (Siltronix) using a HF:H_2_O:EtOH solution with a 15:15:70 proportion, respectively, and a current density of −50 mA/cm^2^ [24].

The electrochemical experiments were performed at room temperature using an AUTOLAB PGSTAT302N (Metrohm, Switzerland) potentiostat/galvanostat equipped by a frequency response analyzer controlled with the NOVA software. A hand-made three-electrode cell (*V* = 5 ml) made by a cylindrical Teflon chamber (base diameter = 2.5 cm; height = 5 cm) containing the electrolytic solution was used. The PSi working electrode was located at the bottom of the cell, and the electrical contact consists in an aluminum disc. A platinum grid placed in front of the anode at 1 cm distance constituted the counter electrode. A platinum tip was used as a quasi-reference electrode. The exposed geometrical area of the electrodes was 0.5 cm^2^ (diameter = 0.7 cm).

The functionalization of PSi substrates was performed both by cyclic voltammetry (CV) and potentiostatic techniques in ACN + 0.1 M (TBAPF6) solutions containing 2 mM of 4-NBD. Consecutive CVs in the potential range −0.4/−1.2 V at a scan rate of 10 mV/s were performed with a number of cycles of 2, 5, 8 and 12. The potentiostatic runs were performed by applying −1.1 V for 600 s.

The modified substrates were characterized by electrochemical impedance spectroscopy (EIS) in ACN solution containing 0.1 M (TBAPF6) by varying the frequency from 30 kHz down to 0.1 Hz at open circuit potential (OCP) with excitation amplitude of 10 mV. The impedance spectra were then fitted to an equivalent electrical circuit by using the ZSimpWin 2.0 software (EChem software).

### Optical Reflectivity

Optical reflectivity measurements were performed by using a PerkinElmer Lambda 950 UV/Vis/NIR Spectrophotometer equipped with a Universal Reflectance Accessory.

### Optical Lithography

The lithographic procedure was performed through a positive process. In the first step, the AZ5214E photoresist was spin coated in a thin and uniform layer on the sample surface. Later, a soft baking was performed in order to remove most of the solvent from the resist coating, making it photosensitive.

By means of a Karl Suss mask aligner in contact printing mode, a matrix of 50 × 50 μm^2^ metallic squares was patterned on the samples, exposing their resist-coated surface to a high-intensity UV light. After exposure, the photoresist was developed in a solution 1:1 by volume of commercial sodium-based AZ developer and deionized water.

Therefore, a 5-nm Ti thin film was electron beam evaporated as buffer layer to improve the adhesion of an upper 90-nm Au layer as metallic contact.

## Results and Discussion

### Grafting Process

**Fig. 2:**
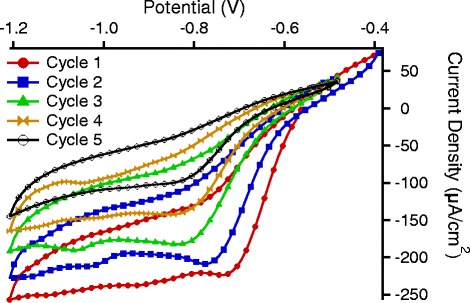
Example of current density vs. potential for five cyclic voltammograms used for grafting process

Figure 2 reports the cyclic voltammograms recorded during the electrodeposition of 4-NBD on PSi surface in ACN containing 2 mM 4-NBD and 0.1 M (TBAPF6) as supporting electrolyte. The current density was calculated with respect to the external surface of the sample. As shown, an irreversible diazonium reduction peak is present in the first cathodic scan at −0.7 V, indicating the cleavage of dinitrogen and the subsequent one electron covalent reaction between the silicon and nitrobenzene radicals. As the number of cycles increases, the reduction peak becomes less evident and a shift towards negative potentials is observed.

As previously reported in the literature [19, 25, 26], the modification of monocrystalline H-terminated silicon can be recognized by a well-defined reduction peak during the first voltammetric cycle indicating a fast and easy reduction of aryl diazonium cations to aryl radicals which can graft on surface. In the second scan, the peak disappears nearly completely and this is indicative of the blocking effect of a dense monolayer which hinders the further reduction at the surface. Instead, in our case, given the large developed surface of PSi, the diazonium reduction peak is still visible after five cycles indicating that the density of grafted molecules is not high enough in the first cycle. In fact, the porous structure of the silicon offers a very large surface and during subsequent cycles, further electron transfer is possible. The negative shift of the reduction peak can be attributed to exchange of electrons between the silicon and diazonium molecules that are reduced on the already grafted molecules allowing the formation of a multilayer structure.

**Fig. 3:**
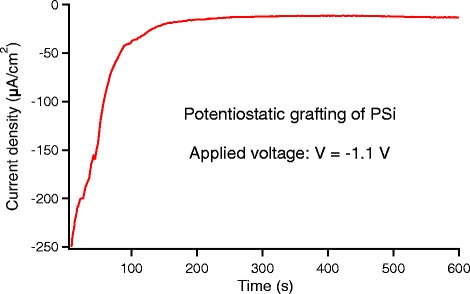
Example of current density vs time for potentiostatic method used for grafting process

Figure 3 shows the time evolution of the potentiostatic current during the 4-NBD deposition. The applied constant voltage was −1.1 V. As can be seen, current intensity is reduced by about one order of magnitude after about 2 min indicating a complete passivation of the surface.

**Fig. 4:**
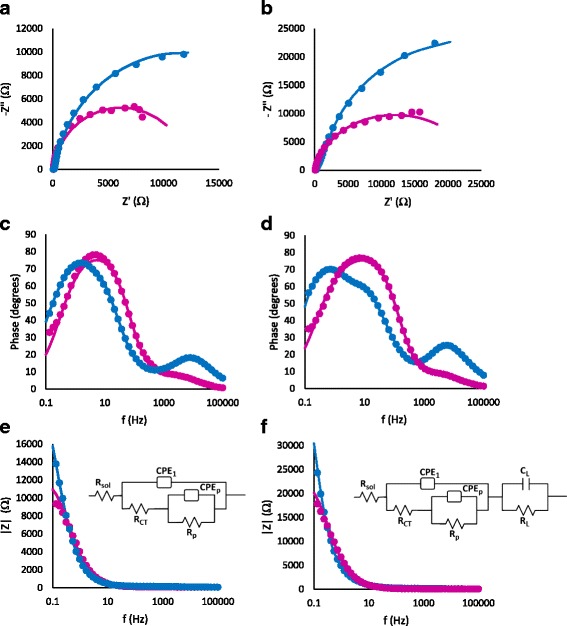
Nyquist and Bode plots for pristine PSi (*purple*) vs grafted PSi (*blue*) in the case of modification using potentiostatic method (**a**, **c**, and **e**) and in the case of CV (**b**, **d**, **f**)

In order to gain information about the nitrophenyl layer, whose thickness can be estimated as 1–2 nm from the reduction of the average pore opening diameter in the case of 12 CV cycles samples, electrochemical impedance spectroscopy measurements have been performed at OCP in a 4-NBD-free solution. Figure 4 shows the Nyquist and Bode plots for PSi modified using five voltammetric cycles (Fig. 4b, d, f) and potentiostatic mode (Fig. 4a, c, e): in both cases, the deposition of the organic layer produces a significant increase of the impedance (blue curves) with respect to unmodified pristine PSi (purple curves). As shown in the Bode phase plots, the modification of the silicon surface allows the increase of the phase angle at high frequencies for both deposition techniques, being higher for the sample grafted with five CV cycles.

The second phase maximum present in pristine PSi is shifted towards lower frequencies after the grafting process: moreover, the sample modified by cyclic voltammetries shows a new overlapped wave indicating the presence of a new time constant. In order to obtain the characteristic parameters of the electrodes, the impedance spectra of unmodified PSi have been modeled with the equivalent circuit proposed for porous silicon electrodes (see inset in Fig. 4e).

The following elements have been inserted in the circuit: the solution resistance (*R*_S_), a parallel combination of the charge transfer resistance (*R*_CT_) and a constant phase element (CPE) instead of double layer capacitance (*C*_dl_) for taking into account roughness effects and variation in the composition of the surface. The CPE exponent, *n*, is generally associated to the surface imperfections [27] The second parallel combination (*R*_p_ and *C*_p_) has been introduced to account for the resistance and capacitance of the porous layer. The impedance spectra obtained for PSi modified by potentiostatic mode have been modeled with the same equivalent circuit, but using a CPE for the porous layer while for the sample modified with five CV cycles, a third parallel combination has been added (see inset in Fig. 4f). *R*_L_ and *C*_L_ elements can be related to the presence of an organic layer with high degree of coverage of the overall surface.

A good fit of the impedance spectra (*χ*^2^ < 8 × 10^−4^) has been obtained with these equivalent circuits.

The fitting parameters are presented in Table 1: *R*_CT_ increases after the grafting process, while the capacitance of the CPE element decreases indicating an increase in the thickness of the electronic double layer. Both the effects can be related to the presence of the phenyl layer on the surface. The exponent of the constant phase element associated to the pores (*n*_*p*_) is indicative of the presence of inhomogeneity or variation in the composition of surface inside the pore: *n*_*p*_, for the sample modified by potentiostatic mode, approaches to 1, which can be related to a poor modification inside the pore structure, while for the sample modified by five CV cycles, we obtain *n*_*p*_ << 1 (see Table 1). Indication of a higher degree of coverage in the surface inside and outside the porous structure is given by the RC parallel combination required to model the sample modified by five CV cycles.

**Table 1:** Computed values obtained by simulation of impedance data by equivalent circuit analyses for PSi samples before and after modification both with potentiostatic mode (sample 1) and five CV (sample 2)

		*R*_sol_ (Ω)	*C*_dl_ (μF)	*n*	*R*_ct_ (Ω)	*C*_p_ (μF)	*R*_p_ (kΩ)	*n*_*p*_	*R*_L_ (kΩ)	*C*_L_ (μF)
Sample 1	Pristine PSi	55.38	3.38	0.78	30.18	24	12.4	–		
	PSi pot.	56.9	0.45	0.72	103.3	57	23.6	0.91		
Sample 2	pristine PSi	55.62	1.90	0.76	29.6	11.2	27.4	–		
	PSi 5 CV	61.13	0.34	0.81	146.8	45.9	1.88	0.77	45.8	42.5

### Optical Reflectivity

Functionalized PSi layer was immersed in NaOH aqueous since it is well known that the dissolution of pristine PSi after immersion in a NaOH solution takes place very quickly [7, 28], so we used concentrated aqueous solutions of NaOH to test the chemical resistance of our grafted samples. We used two solutions with two different concentrations: 0.5 and 0.1 M. The sample dipping step duration was 10 s for the first two steps and 2.5 to 5 min for the other steps, depending on the samples chemical resistance.

In order to characterize the improved resistance of grafted samples to alkaline etching, we monitored the optical reflectivity after each etching step. The reflectivity spectra were measured in the 250–2500 nm wavelength range. Optical reflectivity is a very sensitive technique for the porous layer modifications since PSi, given the pore diameter of 10–20 nm, is seen by the light as a homogeneous layer in the wavelength interval we used. Any modification of the PSi layer, then, would lead to a modification of its overall refractive index and then of the thin-layer interference fringes. Observing the variations in the thin-layer interference fringes amplitude and position, together with the Si reflectivity peaks, it is possible to follow the modifications of the layer with respect to initial freshly grafted PSi layer.

**Fig. 5:**
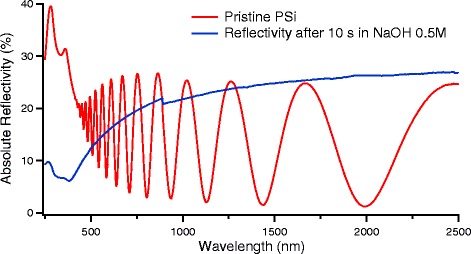
Evidence of the rapid corrosion process of PSi in NaOH 0.5 M aqueous solution

In Fig. 5, as a reference, we show the effect on a pristine PSi layer of a 10-s dipping in a 0.5-M NaOH aqueous solution. The red curve represents the porous silicon reflectivity, and the typical Fabry-Pérot fringes related to the thin-layer interferences are clearly visible for wavelengths longer than about 400 nm. After 10 s dipping (blue curve), as expected, the interference fringes are no more visible due to the almost complete dissolution of the porous layer.

The behavior of grafted PSi samples is markedly different. During the initial dipping, it was possible to observe by naked eye that the portion of the PSi layer outside the nitrobenzene-grafted area (that is the external surface region near the bulk Si—porosified Si separation border) was immediately dissolved while the grafted area appeared unmodified throughout the process.

To characterize the resistance of the layers, as for the pristine PSi layer, we used optical reflectivity measurements. For all samples, we measured the reflectivity of both pristine and grafted layers, as a reference.

**Fig. 6:**
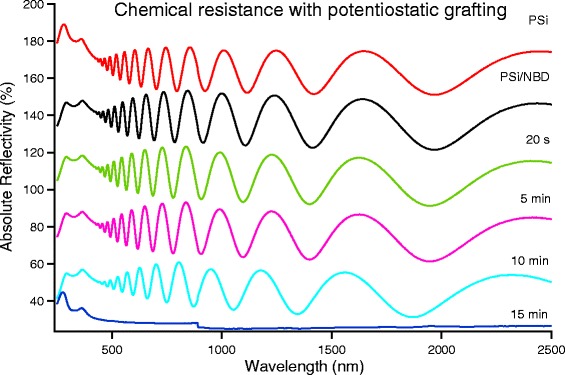
Evolution from the reflectivity of PSi grafted with 4-NBD by potentiostatic method and immersed in NaOH 0.5 M. The spectra, to ease their comparison, are vertically shifted with respect to each other by a 30 % amount. The bottom spectrum has no vertical shift

In Fig. 6, we show the evolution of the reflectivity as a function of the total dipping time for a PSi sample whose grafting has been realized using the potentiostatic method. The pristine and freshly grafted samples are also shown (red and black curves, respectively). The decreased reflectivity of the Si-related double peak and the interference fringes displacement demonstrate both the Si covering and the modification of the average refractive index of the porous layer induced by the nitrobenzene grafting.

In Fig. 6 we observe that the spectrum shape remains unmodified after about 5 min of NaOH dipping. For comparison, it is worth noting that the portion of PSi layer not covered by diazonium was immediately dissolved in the alkaline bath while the covered area stayed stable for much longer in the process. After 10 min, the reflectivity started to change, and after 15 min, dipping no trace of the porous layer is observed, indicating a full PSi dissolution. The potentiostatic grafting method has clearly improved the chemical resistance of the PSi layer that remains unmodified for two order of magnitude longer times with respect the pristine porous silicon.

Using the same scheme, we studied the behavior of samples whose grafting has been performed by cyclic voltammetry. Since the PSi coverage depends on the number of CV cycles (as shown in Fig. 2), we tested the chemical resistance of the samples grafted using different numbers of CV cycles.

**Fig. 7:**
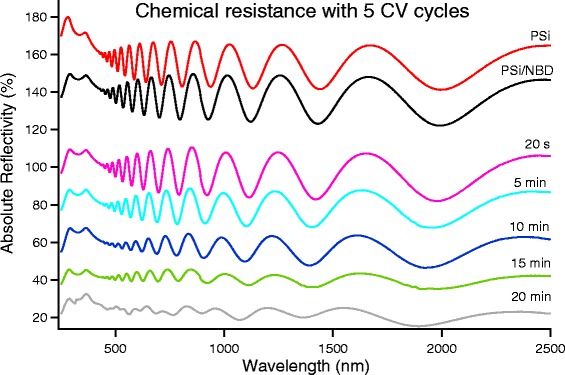
Evolution from the reflectivity of the pristine PSi compared with PSi grafted with five CV cycles and immersed in NaOH 0.5 M. The spectra, to ease their comparison, are vertically shifted with respect to each other by a 20 % amount. The bottom spectrum has no vertical shift

In Fig. 7, we show the evolution of the reflectivity spectra of a PSi grafted with 5 CV cycles as a function of the dipping time. We observe a significant increase by a factor of 2 to 4 with respect the functionalization in potentiostatic regime: after 20 min etch, while clearly partially degraded, the thin film interference fringes were still visible, indicating that the porous layer was still present.

Since, as shown in Fig. 2, after five CV cycles the reduction peak is still present, we performed the chemical resistance test on samples modified with different numbers of CV cycles. For less than five CV cycles, the chemical resistance, while still higher than that observed for potentiostatic grafted samples, was decreased with respect to that obtained for the sample of Fig. 7. For more than five CV cycles, instead, an increase of the chemical resistance is observed.

**Fig. 8:**
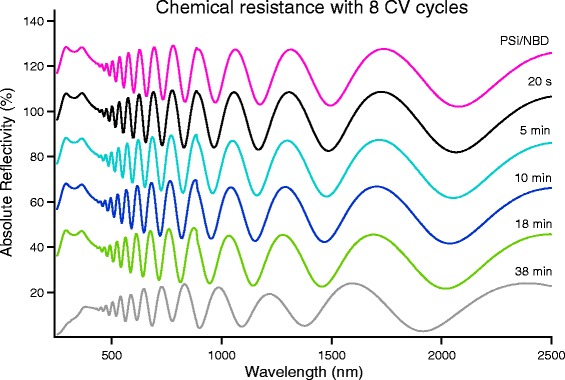
Evolution from the reflectivity of PSi grafted with eight CV cycles and immersed in NaOH 0.5 M. The spectra, to ease their comparison, are vertically shifted with respect to each other by a 25 % amount. The bottom spectrum has no vertical shift

In Fig. 8, we show the evolution of the reflectivity spectra, as in Fig. 7, for a PSi sample grafted with eight CV cycles. In this case, it is clear that there is a significant improvement with respect to the five CV cycles grafting: after 38 min, the PSi structure is only slightly modified. Further increasing the number of cycles does not improve significantly the chemical resistance, even if the reduction peak is visible up to the 12th cycle.

### Optical Lithography Process and Further Characterizations

A morphological characterization of porous silicon after 4-NBD functionalization has been performed through scanning electron microscopy.

**Fig. 9:**
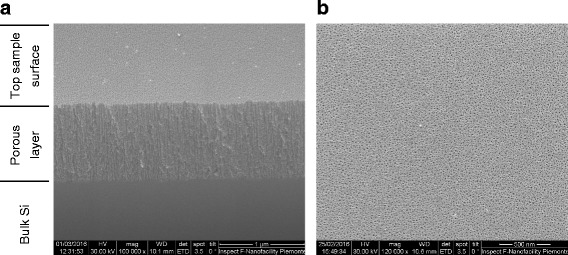
SEM images of cross section (**a**) and plane view (**b**) of a porous silicon sample 4-NBD-grafted with 12 CV cycles. The different parts of the sample in **a** are indicated

The sample cross-section shown in Fig. 9a demonstrates that the pore openings are still clearly visible and there is no evidence of pore clogging (Fig. 9b). It is worth noting that the sample of Fig. 9 has been functionalized using 12 CV cycles; the number of cycles for which the diazonium reduction peak has almost disappeared. This is an important feature, since after the electrochemical processing, there is still room for further functionalization and/or pore filling with other materials.

The electrical resistance of the grafted samples was tested by using the electrical metallic pads patterned on the outside surface by optical lithography and lift-off technique and compared with the response of the pristine PSi.

**Fig. 10:**
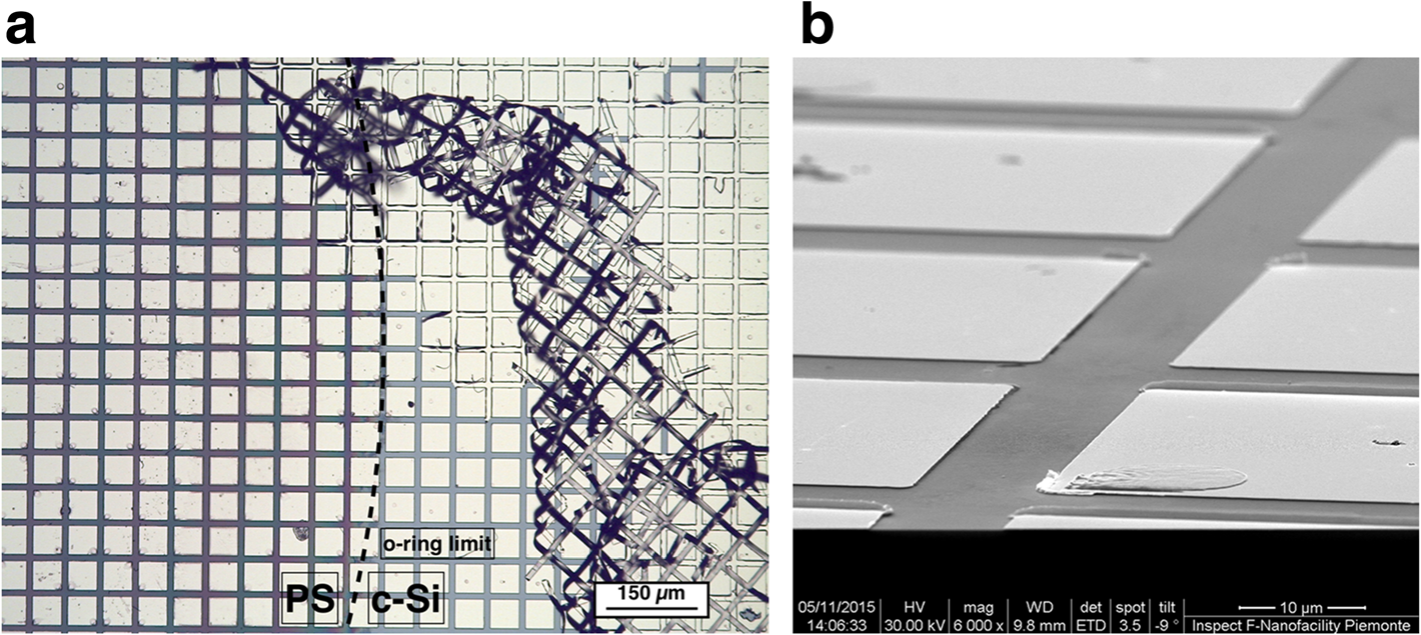
**a** Optical microscope image of the matrix of gold electrical contacts and the metallic grid in excess being removed with acetone; **b** SEM tilted-view of the metallic pattern (FEI Inspect-F images)

In Fig. 10, we show optical and scanning electron microscope (SEM) images of the samples in the final lithographic steps. In Fig. 10a, we show the optical microscopy image of the region across the o-ring separating the area in contact with the acetone bath (left side) with the outside area (right side). The residual photoresist lifting is clearly visible on the right side. The final matrix of gold electrical contacts is shown in the SEM image of Fig. 10b.

**Fig. 11:**
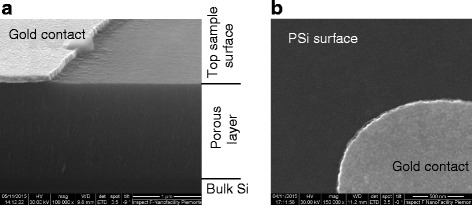
SEM image of the cross section of the porous silicon layer (**a**) and of its surface (**b**) after the 4-NBD functionalization and photolithographic process. The porous layer is fully preserved, and the silicon opened pores are still visible (FEI Inspect-F images). The different parts of the samples are indicated

After the lithographic process, the patterned samples were visually inspected by SEM to determine the effectiveness of the 4-NBD deposition on the protection of silicon porous layer from NaOH-based alkaline developing solution. The SEM images revealed a fully preserved porous layer (Fig. 11). In particular, the porous structure is clearly visible in the cross section (Fig. 11a) as well as the open pores in the top view (Fig. 11b). This demonstrates that the 4-NBD functionalization of the PSi is an effective way to protect the silicon surface from the chemical attack by the alkaline solution.

**Fig. 12:**
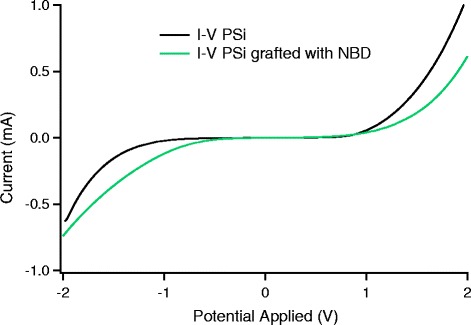
Current–voltage characteristics of a grafted (*green curve*) and pristine (*black curve*) PSi sample. The interdigitated contact area was 3.4 mm^2^

In order to validate the employment of 4-NBD in the porous silicon functionalization, we performed electrical measurements acquiring current–voltage characteristics of the pristine PSi comparing it to the response related to the 4-NBD-functionalized PSi using interdigitated contacts. The results are shown in Fig. 12, and the comparison of pristine PSi (black curve) and grafted PSi (green curve) does not highlight any relevant differences in the trend of the curves enforcing the 4-NBD functionalization process. The only difference between the two systems is a slight variation of the resistance in the treated sample.

## Conclusions

We successfully patterned a matrix of electrical contacts on porous silicon using standard optical lithography on samples treated with 4-NBD. The 4-NBD was deposited using CV and potentiostatic methods. This coating dramatically increases the chemical resistance of the PSi layers to harsh alkaline solutions: instead of a few seconds, the samples stay unmodified up to about 40 min. Moreover, the pores being still open, it is possible to further functionalize the samples for a large variety of applications that requires stable substrates. CV grafting demonstrates significant improvement on the achieved chemical resistance over the potentiostatic grafting method, multiplying by at least a factor of four the maximum dipping time. This result not only demonstrates that this coating protects the PSi surface from alkaline solutions but also opens the way for efficient pore filling with conducting polymers of interest for energy conversion and bio-sensing. Moreover, the lithography of contacts allows tailored electrical characterization of the resulting devices.

## Abbreviations

ACN: Anhydrous acetonitrile
C_dl_: Double layer capacitance
CPE: Constant phase element
CV: Cyclic voltammetry
EIS: Electrochemical impedance spectroscopy
4-NBD: 4-Nitrobenzenediazonium
OCP: Open circuit potential
PSi: Porous silicon
R_CT_: Charge transfer resistance
R_s_: Solution resistance
SEM: Scanning electron microscope
TBAPF6: Tetrabutylammonium hexafluorophosfate
